# Gene Expression Signatures Associated With Immune and Virological Responses to Therapeutic Vaccination With Dendritic Cells in HIV-Infected Individuals

**DOI:** 10.3389/fimmu.2019.00874

**Published:** 2019-04-24

**Authors:** Rodolphe Thiébaut, Boris P. Hejblum, Hakim Hocini, Henri Bonnabau, Jason Skinner, Monica Montes, Christine Lacabaratz, Laura Richert, Karolina Palucka, Jacques Banchereau, Yves Lévy

**Affiliations:** ^1^Inserm, Bordeaux Population Health Research Center, UMR 1219, Univ. Bordeaux, ISPED, Bordeaux, France; ^2^INRIA, SISTM, Bordeaux, France; ^3^VACCINE RESEARCH INSTITUTE – VRI, Groupe Henri-Mondor Albert-Chenevier, Créteil, France; ^4^INSERM, Unité U955, Créteil, France; ^5^Université Paris-Est, Faculté de Médecine, UMR-S 955 Créteil, France; ^6^Baylor Institute for Immunology Research, Ralph Steinman Center for Cancer Vaccines, Dallas, TX, United States

**Keywords:** dendritic cell, HIV, antiretroviral therapy interruption, therapeutic vaccine, gene expression, systems biology

## Abstract

The goal of HIV therapeutic vaccination is to induce HIV-specific immune response able to control HIV replication. We previously reported that vaccination with *ex vivo* generated Dendritic Cells (DC) loaded with HIV-lipopeptides in HIV-infected patients (*n* = 19) on antiretroviral therapy (ART) was well-tolerated and immunogenic. Vaccine-elicited HIV-specific T cell responses were associated with improved control of viral replication following antiretroviral interruption (ATI from w24 to w48). We show an inverse relationship between HIV-specific responses (production of IL-2, IL-13, IL-21, IFN-g, CD4 polyfunctionality, i.e., production of at least two cytokines) and the peak of viral load during ATI. Here we have performed an integrative systems vaccinology analysis including: (i) post vaccination (w16) immune responses assessed by cytometry, cytokine secretion, and Interferon-γ ELISPOT assays; (ii) whole blood and cellular gene expression measured during vaccination; and (iii) viral parameters following ATI, with the objective to disentangle the relationships between these markers and to identify vaccine signatures. During vaccination, 69 gene expression modules out of 260 varied significantly including (by order of significance) modules related to inflammation (Chaussabel Modules M3.2, M4.13, M4.6, M5.7, M7.1, M4.2), plasma cells (M4.11) and T cells (M4.1, 4.15). Cellular immune responses were positively correlated to genes belonging to T cell functional modules (M4.1, M4.15) at w16 and negatively correlated to genes belonging to inflammation modules (M7.1, M5.7, M3.2, M4.13, M4.2). More specifically, we show that prolonged increased abundance of inflammatory gene pathways related to toll-like receptor signaling (especially TLR4) are associated with both lower vaccine immune responses and control of viral replication post ATI. Further comparison of DC vaccine gene signatures with previously reported non-HIV vaccine signatures, such as flu and pneumococcal vaccines, revealed common pathways across vaccines. Overall, these results show that too long duration and too high intensity of vaccine inflammatory responses hamper the magnitude of effector responses.

## Introduction

Systems biology approaches applied to immunology and vaccinology aim at analyzing the whole data available from various high throughput technologies to better understand and predict diseases and mechanisms of interventions (1–3). In vaccinology, these approaches have been successfully used in several applications such as yellow fever (4), influenza (5–9), malaria (10), pneumococcus and meningococcus (11, 12), and HIV vaccines (13) to identify gene signatures associated with vaccine responses. This is exemplified by early changes of expression of some genes following Yellow Fever vaccination, such as TNFRSF17, which represent a signature predictive of cellular and humoral responses to this vaccine (4). In the same line, innate immune pathways associated with responses to rVSV-ZEBOV Ebola vaccine have been identified recently (14). These studies of changes in gene expression of components of the innate immune system help to identify relationships between various pathways involved in responses to vaccines (15, 16) and may contribute to understand variations in vaccine responses according to individual characteristics such as the age (17), sex (6), or seasons (18). There is also a recent trend toward comparing the signatures of various vaccines to distinguish common and specific pathways modulated by each vaccine (11, 12).

After more than 25 years of developing HIV therapeutic vaccines, overall the clinical effects of candidate vaccines remain disappointing [review in (19)]. Despite the capability of eliciting strong immune responses, the ability of the candidate vaccines to fully control HIV replication following antiretroviral treatment interruption remains modest and inconsistent across trials (20, 21). One of the major obstacles for therapeutic HIV vaccine development is the lack of clear understanding of mechanisms of action of the majority of the candidate vaccines and of immune correlates of HIV control. Here, we take the opportunity to apply a systems vaccinology approach to integrate virological, immunological and transcriptomic data from a clinical trial of vaccination with *ex vivo* generated Dendritic Cells (DC) loaded with HIV-lipopeptides in HIV infected patients on antiretroviral therapy (ART). The primary report of this study (DALIA study) showed that the vaccination strategy was well-tolerated and immunogenic (22). Vaccine-elicited HIV-specific T cell responses were associated with improved control of viral replication following antiretroviral interruption (ATI). Here, we report results from an integrative analysis taking advantage of repeated sampling and a large array of immuno-monitoring assays including whole blood and cellular gene expression, as well as phenotypic and cytokine production in response to HIV vaccine antigens. We show here correlations between gene signatures, cellular responses measured before ATI and the magnitude of HIV rebound following ATI in vaccinated individuals. Especially, inflammatory pathways linked to TLR 4 were associated with poor vaccine responses whereas T cell modules induced by the vaccination were associated with viral control. Finally, by applying new statistical tools (23, 24) to the re-analysis of existing data on the responses to other vaccines (11), we reveal common pathways associated with the response to different vaccines.

## Methods

### DALIA Phase 1/2 Trial

The ANRS/VRI DALIA 1 is a phase I single-center study (North Texas Infectious Diseases Consultants, Dallas, TX) sponsored by the Baylor Institute for Immunology Research and the Agence Nationale de Recherches sur le SIDA et les hepatites (INSERM ANRS). The study was approved by the IRB of Baylor Research Institute (BRI) (NCT 00796770). All patients gave written informed consent.

**Figure 1:**
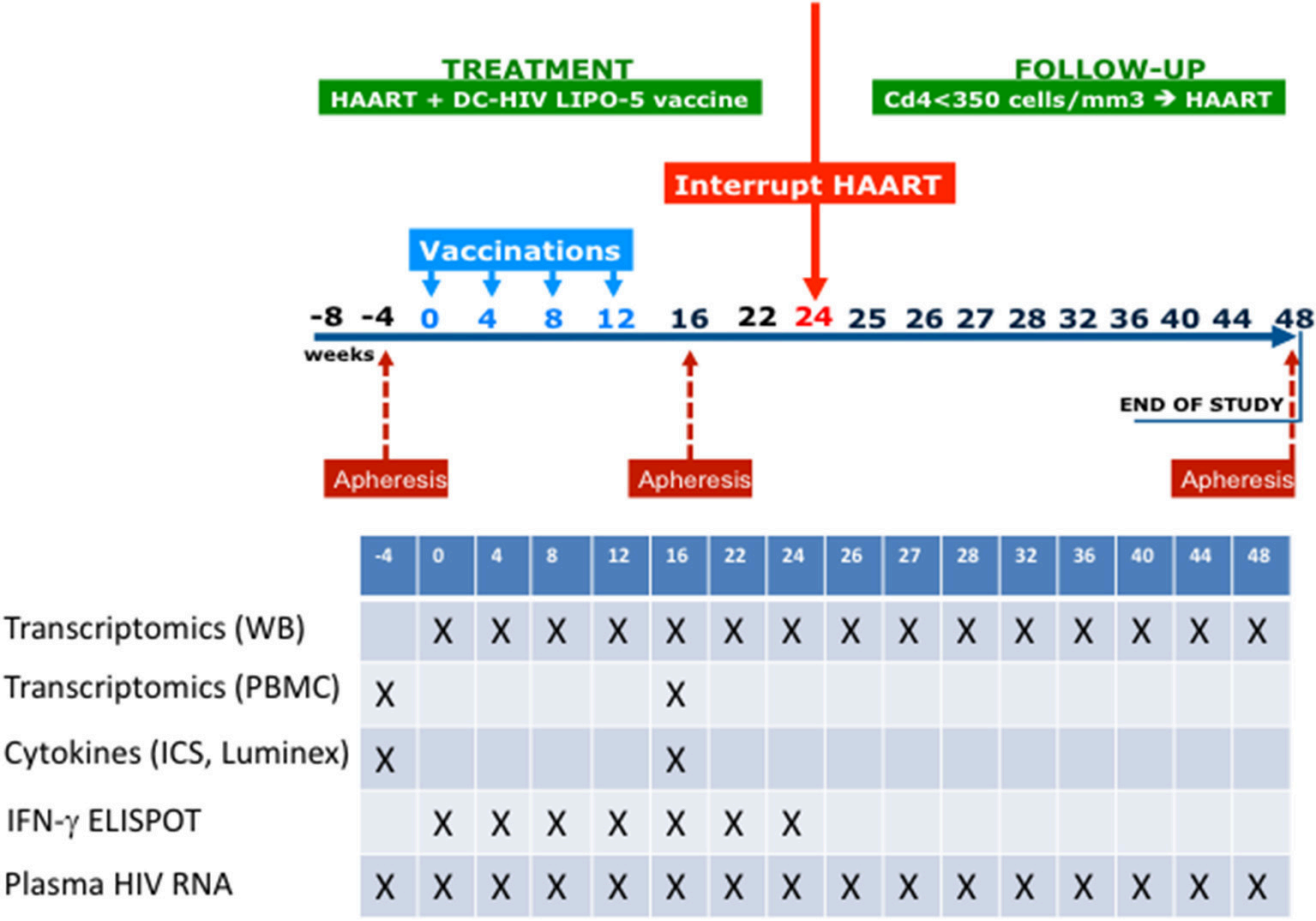
Outline of the ANRS/VRI DALIA 1 clinical trial. Gene abundance in whole blood has been evaluated at any single time point. Gene abundance following PBMC isolation and stimulation has been done with sample coming from baseline (W-4) and after vaccination (W16). At W16, a deep immunological evaluation has been performed with Multiplex, intracellular staining and ELISPOT.

The study design is shown in Figure 1. Eligible patients were asymptomatic HIV-1-infected adults with CD4+ T cell counts >500 cells/μL, CD4+ T cells ≥25%, plasma HIV RNA <50 copies/mL at screening and within the previous 3 months while on ART, with CD4+ nadir ≥300 cells/μL, and no history of AIDS-defining events. Nineteen patients were enrolled.

Participants received four vaccinations at w0, 4, 8, and 12. At w22, patients who had HIV-1 RNA <400 copies/mL were proposed to interrupt ART from w24 to w48. ART could be resumed from w24 to w48 at any time according to the following criteria: (i) if the patients or their doctors wished so; (ii) if CD4+ T cell count was <350 cells/μL and <25% of total lymphocytes at two consecutive measurements 2-weeks apart; and (iii) in the case of occurrence of an opportunistic infection or a serious non-AIDS defining event.

Post vaccination (w16) immune responses assessment by cytometry, cytokine secretion, and Interferon-γ ELISPOT assays were described elsewhere (22).

### RNA Isolation and Microarray Sample Preparation

Whole blood RNA was purified using *Tempus*™ Spin RNA Isolation *Kit* (ThermoFisher scientific). PBMC, CD4- and CD8-lymphocytes RNA were purified on Qiagen RNeasy Micro Kit. RNA was quantified using a ND-8000 spectrophotometer (NanoDrop Technologies, Fisher Scientific, Ilkirch Cedex, France) before being checked for integrity on a 2100 BioAnalyzer (Agilent Technologies, Massy Cedex, France). cDNA was synthesized and biotin-labeled cRNA was generated by an *in vitro* transcription reaction using Ambion Illumina TotalPrep RNA Amplification Kits (Applied Biosystem/Ambion, Saint-Aubin, France). Labeled cRNA were hybridized on Illumina Human HT-12V4 BeadChips. All steps were done following the manufacturers' protocols.

### *In vitro* Stimulation of Purified PBMC With HIV Peptides for Gene Expression and Cytokines Profile Analyses

For cytokine profile analysis, *in vitro* stimulation of purified PBMC with HIV antigens has been performed as previously reported (22). For gene expression analysis, 10^6^ of thawed PBMC resuspended in RPMI 1640 media with L-Glutamax supplemented with Penicillin/Streptomycin and 10% HS (R-10HS) were stimulated for 6 and 20 h in 48-well plates with 2 μg/ml of HIV LIPO-5 vaccine itself, a pool of 5 long peptides corresponding to LIPO-5 vaccine sequences (Gag17–35, Gag253–284, Pol325–355, Nef66–97, and Nef116–145) at 2 μM/peptide, or a pool of 36 peptides (15-mers overlapping by 11 amino acids, covering LIPO-5 vaccine sequences) at 2 μg/ml/peptide, in a humidified 37°C cell incubator with 5% CO2. Cells were then transferred in eppendorf tubes, pelleted, resuspended in 350 μL lysis buffer and frozen immediately at −80°C until transcriptomic analysis as described (25).

### Published Data of Whole Blood Transcriptional Response to Influenza and Pneumococcal Vaccines

Data of the whole blood transcriptional response to influenza and pneumococcal vaccines of 46 individuals were publicly available in the NCBI Gene Expression Omnibus under code GSE30101 (11).

### Statistical Analyses

Statistical analyses were performed using R software version 3.2.2 (The R foundation for Statistical Computing, Vienna, Austria). Gene transcription data were pre-processed (25, 26) and corrected for potential batch effects (27). Statistical comparisons between groups of interest were based on empirical Bayes moderated *t-*statistics (28). An adaptive FDR procedure was used to control for test multiplicity (29). Unsupervised hierarchical clustering heatmap analysis was performed on scaled raw expression using Euclidean distance matrix and Ward's linkage method (30). Canonical pathway and biological function analyses were then carried out using genes differentially expressed between groups with adaptive FDR-adjusted *P* < 0.05 and fold-change |FC| >1.5.

Time-course gene set analysis was performed using TcGSA, an innovative approach relying on mixed regression models and likelihood ratio test to identify gene sets the expression which is significantly changing over time (23). It uses pre-defined gene sets, such as the Chaussabel's modules or the BTMs (12). TcGSA accounts for patient heterogeneity and allows gene sets to be broken down into several distinct gene subsets with different dynamics. Gene expression from the DALIA trial was analyzed separately before and after antiretroviral treatment interruption due to the large and noticeable impact of the viral rebound, with a cubic polynomial time-basis for modeling gene-specific non-linear dynamics. Bootstrap analyses were performed to check the consistency of these results and allowed reporting the proportion (among 1,000 bootstrap samples) that a given module was selected. Gene abundances from the studies reported in Obermoser et al. (11) were modeled by a constant expression over time plus a spike at D1 (where the majority of the signal was observed for both the flu and the pneumococcal vaccines).

A transcriptomic signature was then derived through an integrative analysis of the gene expression at w16 from the significant modules identified by TcGSA together with the viro-immunological measurements at w16 in the DALIA trial, performed using sparse group Partial Least Squares (sgPLS) with leave-one-out cross-validation (31). This is a recent variable selection method for high-dimensional data that can account for an essential grouping structure in the data (i.e., the gene sets for the gene expression) and that is based on the maximization of the covariance between two data matrices (namely the viro-immune response and the gene abundances). A regularization approach based on Lasso penalty selects the most contributive variables. This allows to down-select the gene abundances at w16 most associated with the viro-immune response at w16. A complete description of the methods can be found in Liquet et al. (31).

Functional analysis of the immune signature was performed with Ingenuity Pathway Analysis software (IPA®, Qiagen, Redwood City, California, Spring Release March 2018) and CluePedia and ClueGO in Cytoscape (32, 33).

All microarray data is MIAME compliant and the raw and normalized data have been deposited in the MIAME compliant database Gene Expression Omnibus (http://www.ncbi.nlm.nih.gov/geo/, GEO Series accession number GSE46734).

## Results

### Changes in Whole Blood Gene Expression in Individuals Immunized With *ex vivo* Generated DC HIV Vaccine

**Figure 2:**
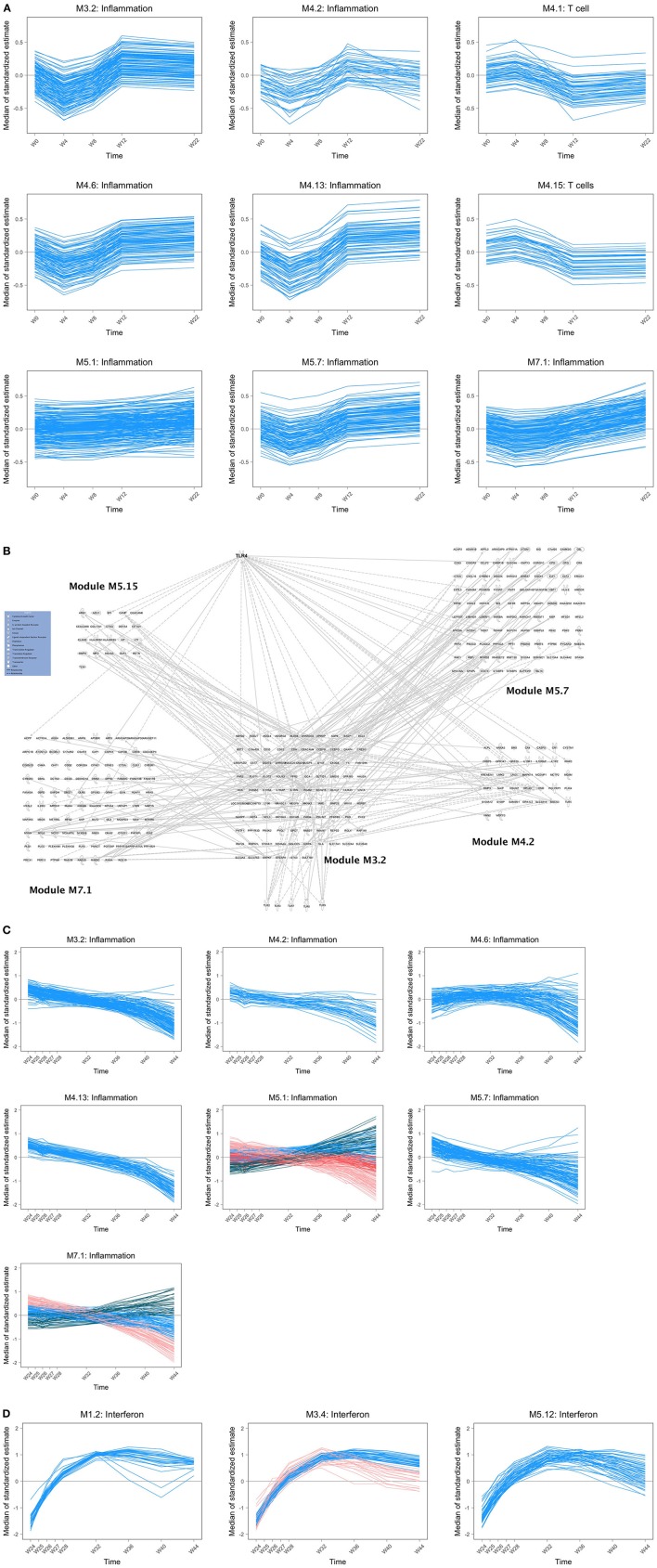
**(A)** Dynamics of gene abundance before antiretroviral treatment interruption. Dynamics of the gene abundance in various modules that changed significantly over time including inflammatory modules (M3.2, M4.13, M4.6, M5.7, M7.1, M4.2, M5.1) and T cell module (M4.1). Lines are smoothed trajectories predicted by the longitudinal statistical model (23). **(B)** Composition of inflammatory modules and relationship with TLRs. Relationships between inflammatory modules M3.2 and M4.2, M5.7, M7.1, and M5.15 (annotated “Neutrophils”) and TLR genes using Pathexplorer in IPA. Links between modules that are not concerning M3.2 are not represented. Links between inflammatory modules other than M3.2 and TLRs other than TLR4 are not represented. **(C)** Dynamics of gene abundance after antiretroviral treatment interruption: inflammatory modules. Dynamics of gene abundance in “inflammatory modules” that changed significantly over time. Various colors reflects different trajectories in the same modules defined by unsupervised clustering (23). **(D)** Dynamics of gene abundance after antiretroviral treatment interruption: interferon modules.

All 19 included patients received the four vaccinations as planned (Figure 1) with no changes in vaccine dose. Among them, 16 had all data available for the integrative analysis. Their characteristics are reported in Table 1. First, we analyzed changes in transcriptomic whole blood gene expression throughout the study; i.e., every 4 weeks from baseline, including time points before each vaccination at weeks 0, 4, 8, and 12 and at week 22, i.e., 2 weeks before ATI. For this, we developed a specific statistical method, the time course gene set analysis, to detect significant change of gene abundance in groups of genes over time taking into account the heterogeneity of the dynamics among a given group (23). A simple gene by gene analysis did not reveal significant changes after correction for test multiplicity (False Discovery rate < 5%). The analysis of the time course of gene sets, as defined by Chaussabel et al. (34), revealed 69 modules with dynamics that changed over time before ATI. As shown in Figure 2A, the modules exhibiting significant changes (as reported by order of significance through the percentile distribution of the statistics) were modules annotated: (i) “inflammation” (M3.2, M4.13, M4.6, M5.7, M7.1, M4.2, M5.1) and; (ii) and T cell activation (M4.1, M4.15). The dynamics of gene abundances over time varied between modules (Figure 2A). Inflammation modules M3.2, M4.2, M4.6, M4.13, M5.7 decreased in average abundance during the period of vaccination and then increased to reach levels above baseline before treatment interruption (at w16). Interestingly, the T cell modules (M4.1, M4.15) presented inverse dynamics. The inflammation modules M5.1 and M7.1 increased continuously during the vaccination period.

**Table 1:** Baseline characteristics.

		***n* = 16**
**Male**	*n* (%)	14 (88)
**Age (years)**	Median (IQR)	45 (36–49)
**Race/Ethnicity**		
White	*n* (%)	11 (69)
Black/African American	*n* (%)	2 (13)
Hispanic/Latino	*n* (%)	3 (19)
**Body Mass Index (kg/m**^**2**^**)**	Median (IQR)	27 (25–28)
(19–25)	*n* (%)	4 (25)
(25–30)	*n* (%)	11 (69)
> = 30	*n* (%)	1 (6)
**Mode of transmission (type of sexual contact)**		
Homosexual/bisexual	*n* (%)	13 (81)
Heterosexual	*n* (%)	3 (19)
**HIV clinical stage**		
A	*n* (%)	15 (94)
B	*n* (%)	1 (6)
**Nadir CD4+ (/mm**^**3**^**)**	Median (IQR)	346 (318–411)
**CD4+ (/mm**^**3**^**) at W-8**	Median (IQR)	711 (635–930)
**CD4+ (/mm**^**3**^**) at W0**	Median (IQR)	647 (545–757)
**HAART**		
With NRTI	*n* (%)	16 (100)
With NNRTI	*n* (%)	14 (88)
With PI	*n* (%)	3 (19)
**Time between the start date of the first HAART regimen and inclusion (years)**	Median (IQR)	10 (6.7–13.7)
**Time between the start date of current HAART regimen and inclusion (years)**	Median (IQR)	2.8 (1–3.8)

The composition of M3.2 was mainly based on genes related to toll-like receptor signaling pathway, especially TLR4 (Figure 2B) and genes related to neutrophil activation involved in immune response (e.g., ADGRG3, ALOX5, BST1, CD55, CD63, CDA, CKAP4, CRISPLD2, FOLR3, FPR2, GCA, ITGAM, LILRA3, MME, MMP25, OSCAR). Ingenuity Pathway analysis (IPA) revealed an interaction of the different inflammatory modules centered on the M3.2 module and the TLR4 pathway (Figure 2B). The module M4.6 that includes MYD88 is enriched in genes related to type 1 and type 2 (IFN^γ^) interferon signaling pathways. The modules M4.13, annotated “activation and cell division of leukocytes,” M5.7 and M7.1 (Figure 2B) are related to cell activation, and so is module M5.1, which is associated to myeloid cells and neutrophils activation (including TYROBP gene related to several processes). Regarding T cell activation modules, changes in gene expression during the vaccination period concerned more specifically the module M4.1, which is composed of genes related to T cell selection (e.g., BCL11B, BCL2, CCR7, CD28) and diversification of TCR (e.g., BCL11B, LEF1, TCF7); and the module M4.15, which is also related to T cell selection (LY9, THEMIS, ZAP70) and differentiation (e.g., CD2, CD27, GPR18, ITK, LCK, LY9, RASGRP1). In addition, other modules related to platelets (M1.1), mitochondrial respiration and stress (M5.10, M5.6, M6.2), erythrocytes (M3.1, M2.3), plasma cells (M4.11) exhibited significant changes during the vaccination period.

Following ATI (from week 24 onwards), changes in gene expression were assessed every week until week 28 and every 4 weeks until week 44. The abundance of the genes in the inflammatory modules tended to decrease over time up to 44 weeks (Figure 2C). The dynamics of the genes from some modules (M5.1, M7.1) was more complex, with subsets of the genes showing distinct dynamics. During this period, there was a significant increase in the abundance of type-1 interferon genes, as previously described in HIV/SIV primary infection (35), and enriched in the module M1.2, M3.4, and M5.12 (Figure 2D).

In a robustness analysis, the analysis of the dynamics of the genes during vaccination (preATI) was performed with the Blood Transcriptional Modules (BTMs) that are other gene sets generated using another approach than Chaussabel et al. (12). Overall, the results were consistent with the initial analysis using the Chaussabel's modules. There was a significant variation of 26 modules enriched in activated dendritic cells/monocytes (BTM64), in monocytes (BTM11.0, BTM118.0, BTM37.1, BTM163), in T cells (BTM7.0, BTM223) and modules annotated “immune activation” (BTM37.0), “T cell activation” (BTM7.1), “TLR and inflammatory signaling (BTM16). The other modules were “blood coagulation” (BTM11.1), “platelet activation” (BTM32.0, M32.1), and 6 modules were not annotated (see Supplementary Table 1).

### Changes in Cellular Gene Expression in Response to HIV Antigens in Vaccinated Individuals

**Figure 3:**
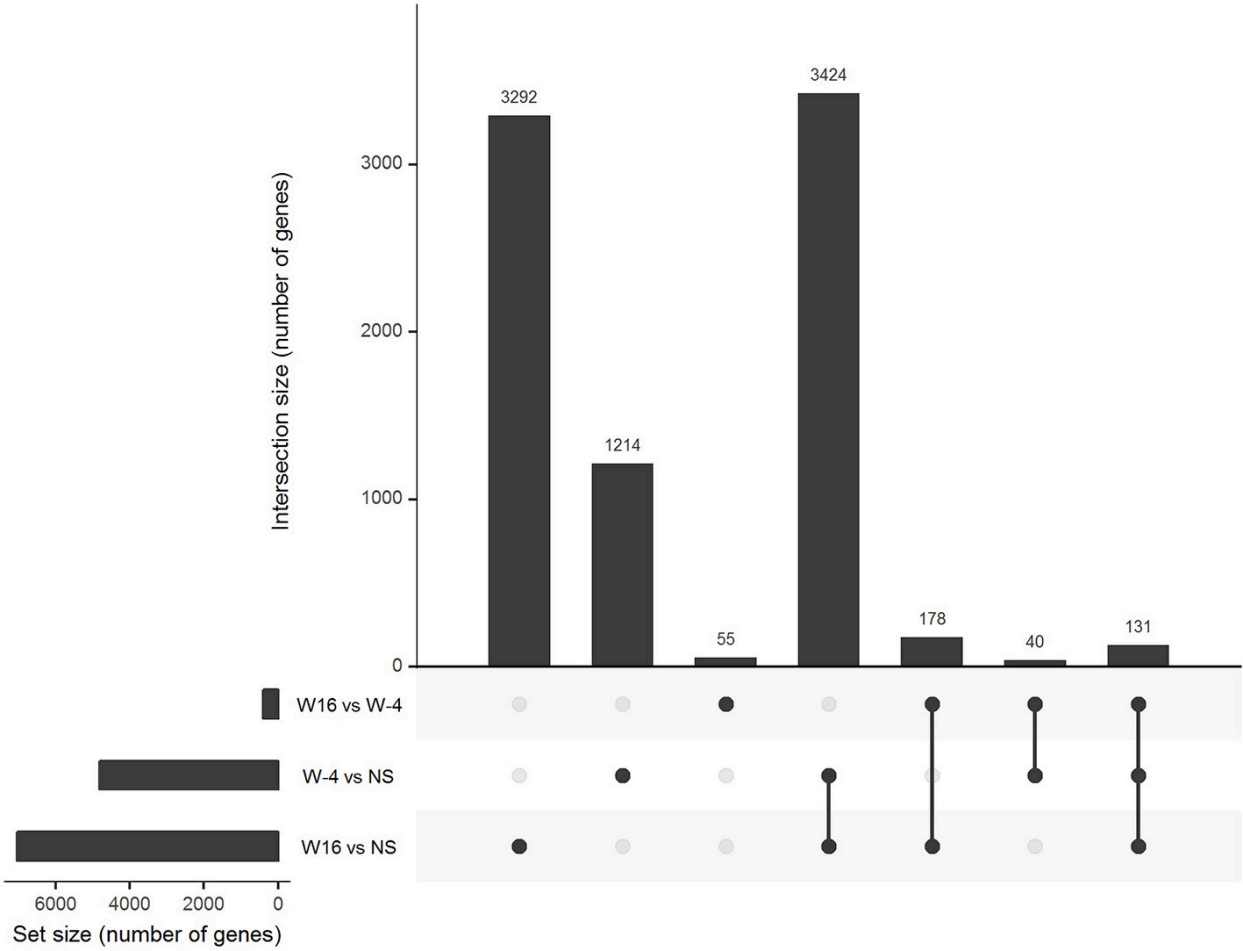
Gene expression in stimulated PBMC. Upset diagram of the genes differentially expressed in PBMC after 20 h of stimulation with 15 mers at week-4 (baseline) and week 16 in comparison of unstimulated cells and between week-4 and week 16.

**Figure 4:**
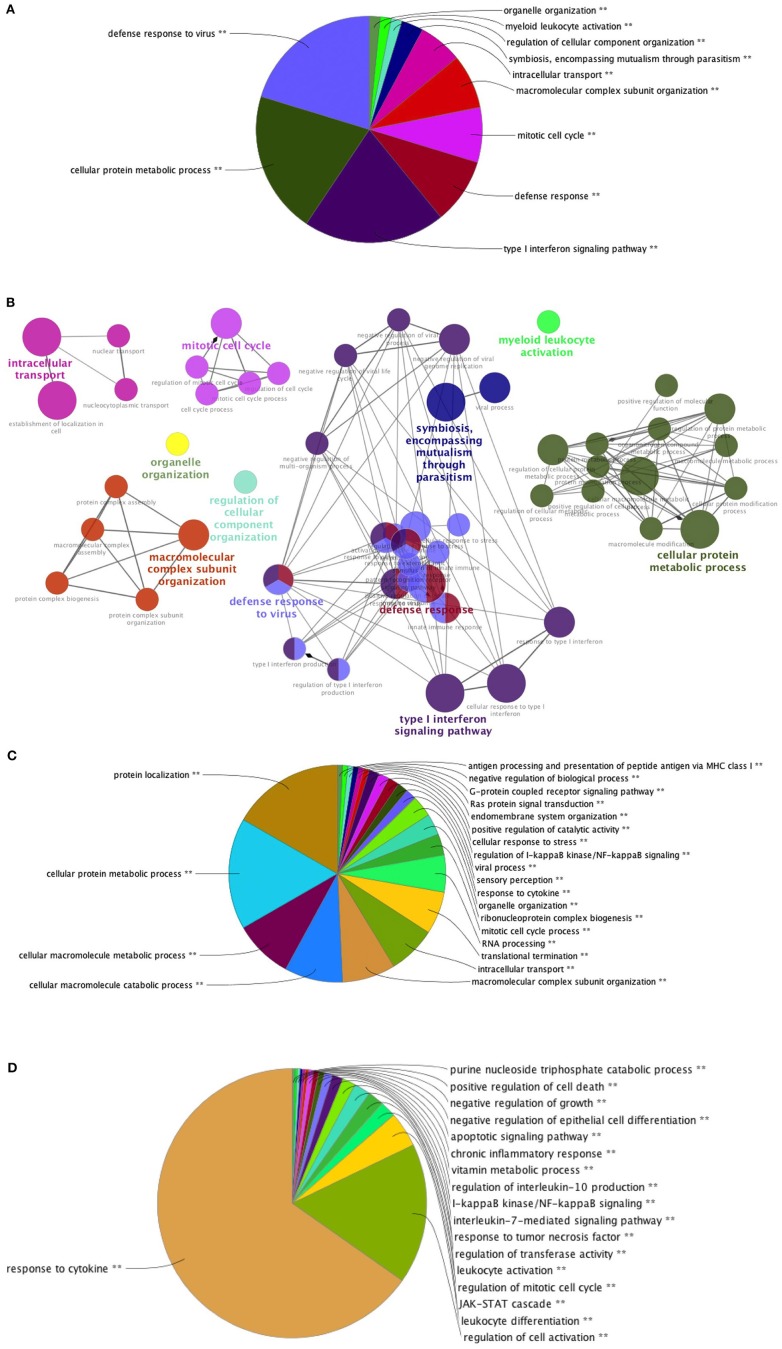
Pathways of differentially expressed genes in stimulated PBMC. Pathways of differentially expressed genes between stimulated and unstimulated cells at: **(A)** W-4 (*N* = 1,214) Overview chart with functional groups and **(B)** Functionally grouped network with terms as nodes linked based on their kappa score **(C)** W16 (*N* = 3,292) and **(D)** genes which expression changed significantly between week-4 (baseline) and week 16 (*N* = 404) after stimulation. Analysis performed with CLUEGO (GO Biological and Immune System processes ontologies, Network specificity: Global).

PBMC collected at baseline and at week 16, 4 weeks after the last vaccination, were stimulated with 15-mers overlapping HIV peptides carried by *ex vivo* DC vaccines (see section Methods) during 20 h. The stimulation by 15-mers led to a differential expression of a large number of genes compared to unstimulated cells (Figure 3). At baseline, the comparison between stimulated and unstimulated conditions showed a differential expression of 4,638 genes, including 1,214 that were differentially expressed at baseline only and not at week 16. The genes differentially expressed at baseline only belonged to various pathways including defense pathways, especially the type 1 interferon signaling pathway (Figures 4A,B). At w16, the comparison revealed a differential expression of 6,716 genes, including 3,292 that were differentially expressed at w16 only. At week 16, even more pathways were mobilized, from general cellular processes to immune response processes (Figure 4C). When looking at the 404 genes that significantly changed between baseline and week 16 after stimulation, there is a clear enrichment of the genes involved in pathways related to response to cytokines: CXCL9, 10, 11, 13, FOXO3, FYN, HLADP, DQ, DR (Figure 4D). More specifically, it involved Th1 and Th2 pathways, T Helper cell differentiation, dendritic cell maturation, and antigen presentation pathways.

### Integrative Systems Vaccinology: Linking Changes in Gene Expression, Vaccine-Elicited Immune Responses, and Viral Dynamics Post ATI

Then, we assessed whether changes in whole blood gene expression identified through module variations at week 16 (4 weeks after the last vaccination and before ATI) were correlated to HIV-specific cellular immune response at the same time point and the viral dynamics following ATI. As previously published (36), the vaccination led to an increase in the magnitude and breath of HIV-specific T-cell responses as measured by IFN-γ ELISPOT after stimulation by HIV peptides pools. HIV-specific CD4+ and CD8+ T responses were polyfunctional (producing 2 or more cytokine) as measured by ICS. Also, vaccination induced a broad repertoire of cytokine-secreting cells as assessed by Luminex assay (IL-2, IL-13, IL-17, IL-21, IP10). The different types of T-cell responses were summarized by a polyfunctionality U-score for multivariate data [see section Methods and reference in (22)]. The cellular responses measured at week 16 after vaccination and before ART interruption were associated with the peak of viral load measured after ATI (22).

**Figure 5:**
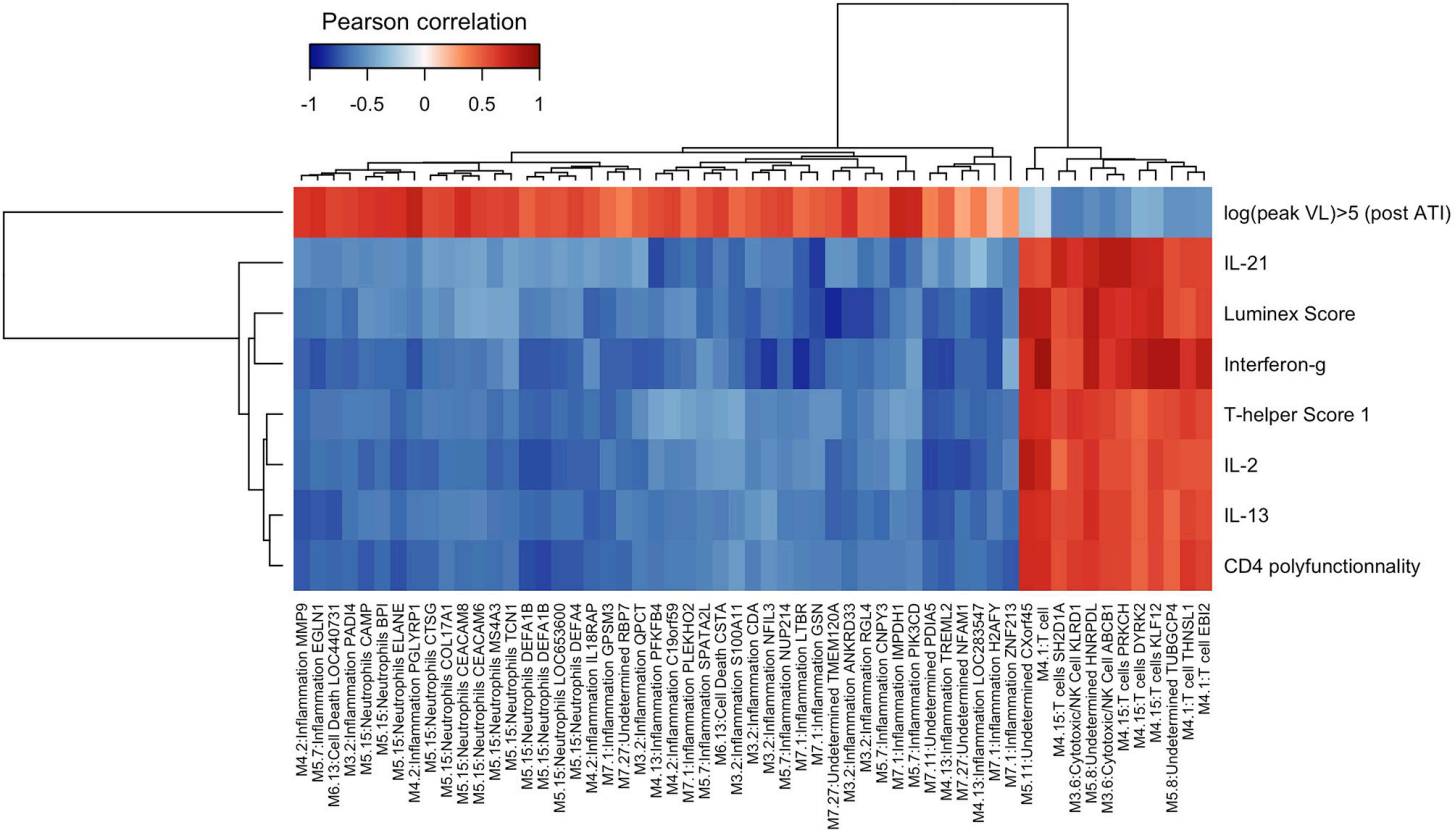
Integrative analysis of changes in gene expressions and cellular immune responses at W16 and viral dynamics after ATI. Correlations from −1 (blue) to +1 (red) estimated from sparse partial least square approach. Peak HIV RNA plasma viral load Post ATI is the maximum observed value of HIV RNA viral load after ATI. Other immune markers have been measured at week 16: IL-21, IFN-γ, IL-2, IL-13 by LUMINEX and CD4 polyfunctionality by ICS. LUMIscore and TH1score are calculated scores using several cytokine measurements at W16 (see section methods).

We found that a large part of the inflammatory modules (M3.2, M4.2, M4.13, M5.7, M7.1) was negatively correlated to vaccine-elicited HIV specific cellular immune responses. The module 5.15, annotated “Neutrophils,” was also negatively correlated to the immune response (Figure 5). All these modules were associated with each other and especially with M3.2 (Figure 2B). In contrast, modules related to the T cell response (M4.1 and M4.15) were positively correlated to these cellular immune responses. Inflammatory gene expression changes were positively correlated with the maximum HIV RNA values post ATI, whereas T cell activation gene expression changes were negatively correlated with this virological parameter (Figure 5). Thus, the patients who mobilized predominantly inflammatory genes in response to vaccination were those who presented the worst immune responses to the vaccine and the highest HIV RNA levels after ATI. The average correlation between gene abundance of inflammation modules and maximum viral load after ATI was 0.53 (Supplementary Figure 1). Interestingly, the inverse correlation identified between inflammatory gene expression and vaccine response at week 16 was already consistently detectable at earlier time points (at weeks 4, 8, and 12) (Supplementary Table 2).

### Highlighting a Common Inflammatory Pathway to Different Vaccines

In order to better characterize mechanisms of dendritic cell-based vaccine immunogenicity, we compared whole blood gene expression signatures identified in our trial with those described with other vaccines, for which protective correlates have been identified. We took the opportunity of published studies in which the same methods for measuring transcriptomic changes were used as the ones in the present study. For this, we revisited published data using our statistical approach based on gene set analyses of longitudinal data [here and detailed in (23)]. Obermauser et al. reported the early commitment of inflammation modules following Pneumococcus and Influenzae vaccinations in healthy humans (11), with a lower inflammatory signal with the Influenzae vaccine. We found a consistent commitment of the inflammatory modules especially M3.2 (including RGL4, CDA, NFIL3), M4.13 (including TREML2), and M4.2 (including S100P) both in our therapeutic HIV vaccine trial and in the Pneumoccocus and Influenzae vaccine data sets (23). Furthermore, an increased abundance of the genes of these modules was associated with a poorer response to the Pneumoccocus vaccine [as reported in (11)] and to the Influenzae vaccine, in accordance with the lower response to dendritic cell-HIV vaccine in our trial.

## Discussion

By taking advantage of both blood and cellular analyses, a longitudinal study design and the comparison between a pre- and post ATI period, we were able to demonstrate an association between the mobilization of inflammatory pathways, the magnitude of vaccine elicited HIV cellular responses and the viral dynamics after ATI after experimental therapeutic DC-based vaccination in HIV-infected patients. The results reported here contribute to identify gene signatures of a therapeutic DC-based vaccine against HIV and to a better understanding of the actions of these vaccines.

Longitudinal analysis of whole blood gene expression revealed changes in gene abundance from modules involved in “inflammation” and “activation” and “differentiation of T cell.” Interestingly, changes in abundance of genes involved in myeloid activation and trafficking were detectable at early vaccination time points. Although activation of inflammatory pathways is known as a physiological response to vaccinations, our results revealed also that modifications of genes related to differentiation and activation of T cells following vaccination behave in the opposite way at the same time points. Consistently, genes related to toll-like receptor signaling pathways and Interferon type 1 and 2 were mobilized in the blood of vaccinated individuals. Comparison between pre- and post-vaccination (w16) changes in gene expression induced by HIV antigen stimulation enriched these observations showing an induction of genes related to cellular immune responses to cytokines, T cell, and dendritic cell responses.

A very specific feature of our study consists in the timing of whole blood sampling for gene expression that was performed every 4 weeks. This schedule is very different from previous studies that usually took blood few hours after vaccination [e.g., (10, 11, 13)]. Here, we thus studied long-term trends of gene abundance that could be associated to changes in equilibrium states of biological functions or changes in the representation of cell populations. The intensity of the signals in the whole blood associated with the vaccinations was high enough to detect the changes of genes abundance and did not require additional analyses taking into account cell populations (37). Analyses at the module (i.e., gene set) level were needed to detect the signals in this study during the vaccination period (23) because this type of analysis is much more powerful than a gene-by-gene analysis (38). By not measuring changes of gene abundance at early days after vaccination, we may have missed the dynamics of the innate response detectable at day 1 and day 3 after vaccination (10, 11, 14, 39). Nevertheless, it is interesting to note that subtle changes are still detectable later on (i.e., after day 7) when using geneset/module approaches with appropriate statistical methods (23). This is confirmed also by our re-analysis of public datasets of transcriptomic changes after Pneumococcus or Influenzae vaccination in humans (11). Modules including genes associated with inflammation (M3.2, M4.2, M5.1, M5.7) were detected day 1 after pneumococcal vaccine but also with flu vaccine when using the same analytical approach than in this paper (11). The same observation was made following Meningococcus vaccine as reported in Li et al. (12). TLR and inflammatory signaling (BTM 16), enrichment in neutrophils (BTM37.1), in monocytes (BTM11.0, BTM118.0) were found 6 h after a vaccination with a recombinant HIV-1 envelop glycoprotein adjuvanted with the TLR4 agonist GLA-AF (13). The same inflammatory/TLR/chemokines BTMs signatures were reported day 1 and day 2 following malaria vaccine based on Ad35 vector or virus-like particles based on a mixture of the fusion reconstruct (RTS) with native HBsAg (S) (10). Therefore, it is interesting to note that signatures mostly reported at early time points were also found later in our study.

A too high and long-standing change in abundance of gene expression associated with toll-like receptor signaling pathways and monocyte/neutrophil activation appeared to be deleterious for the immune response to the vaccine. This may have an important impact on the use of adjuvants that are needed but may require refined adjustments (16) to avoid excessive inflammation that could be deleterious (40). The idea of counter-regulatory mechanisms induced by vaccines has already been discussed such as the role of monocytes limiting the vaccine response (14, 41). This mechanism should be clearly distinguished from the TLR and inflammasome signals that amplify the T cell responses (42) as this last phenomenon occurs at the place of the immune response, that is in the lymph nodes, as an early event in response to vaccine (43). Interestingly, these transcriptional signature in whole blood were also found to be associated to the response to other vaccines such as Pneumococcus (11) and Malaria (10). For instance, in the case of pneumococcal vaccine group, Obermoser et al. reported a negative correlation between serology results and day 7 modular transcriptional data for module associated with inflammation (M3.2, M4.2, and M4.13). The direction of the association between the gene signature and the immunological response may change over time. The cellular CD4-T cell response measured at day 14 after vaccination with Ad35 followed by RTS,S/AS01 was positively associated with the inflammatory/TLR/chemokines responses (including BTM16) at day 1 and negatively at day 6 [see Figure S5 in (10)]. Therefore, the deleterious association of persistent inflammatory signature in whole blood several days after vaccination with the immune response to vaccine has been reported in several vaccine platforms. This cross-validates the phenomenon and indicates a potential broad mechanism driving the immune response to vaccine. Although additional information on single-cell would be of interest to better understand the mechanisms leading to the variation of the vaccine response, key results are biologically relevant and may be further explored by tracing some cell populations such as inflammatory monocytes (41). There are other examples where the transcriptome has helped in deciphering the role of cell populations that was then specifically quantified as in Obermauser et al. (11) where the change of the abundance of genes (e.g., TNFRSF17) that constitutes a plasma cell precursor signature (M4.11) was in line with the dynamics of the concentration of plasmablasts.

The involvement of pathways including TLR4 and MYD88 raises the hypothesis that gut translocation may interfere with the responses to the vaccine as it does with other immunotherapy (44). Other mechanisms could induce TLR4 signaling. It has been established that soluble gp120 has a pro-inflammatory action through induction of TNFα, IL-8, IL-6, CCL2 (45, 46). Inflammatory response can also be triggered by the direct interaction of HIV-gp120 and TLR4 molecule (47). This interaction leads to both the production of proinflammatory cytokines and to increase the HIV replication. Furthermore, TLR expression and function can be up-regulated in response to HIV-1 infection, emphasizing the inflammatory response (48).

These types of result should help improving the potency of vaccines by combining vaccines and immunomodulatory tools (49). For instance, TLR7 agonist has been used successfully to activate the reservoir of SIV-infected monkeys and enhance immunological response of a recombinant adenovirus serotype 26 (Ad26) prime and modified vaccinia Ankara (MVA) boost vaccine (50). However, it is unclear whether the beneficial effect of the TLR7 agonist reflected its potential role as a vaccine adjuvant or as a latency reversing agent. The effect of TLR7 agonist could actually have reduced the immunological effect of the vaccine in case of an exaggerated inflammatory response as described in the present study.

In conclusion, inflammatory pathways related to toll-like receptor signaling pathways that were committed in response to this DC-based therapeutic HIV vaccine were associated with a poorer immune response to vaccination and poorer viral control after ATI. Given a similar involvement and impact of these pathways in responses to other vaccines, it is important to control the kinetics of inflammatory responses induced by vaccines. In total, these results are helpful for the design of further strategies combining vaccines with adjuvants and/or immunomodulators.

## Ethics Statement

The study was approved by the IRB of Baylor Research Institute (BRI) (NCT 00796770). All patients gave written informed consent.

## Author Contributions

YL and JB conceived the project and designed the experiments. HH and JS conducted the transcriptomics analyses. CL and MM conducted the immunological analyses. RT, BH, HB, and LR conducted the statistical analyses. RT and YL analyzed the data and wrote the manuscript. All the authors provided critical feedback on the manuscript prior to publication and have agreed to the final content.

### Conflict of Interest Statement

The authors declare that the research was conducted in the absence of any commercial or financial relationships that could be construed as a potential conflict of interest.
