# Supplementary material for: Gene Expression Signatures Associated With Immune and Virological Responses to Therapeutic Vaccination With Dendritic Cells in HIV-Infected Individuals
Source: Front Immunol. 2019 Apr 24;10:874. doi: 10.3389/fimmu.2019.00874 (PMC6492565; doi:10.3389/fimmu.2019.00874)

Supplementary Material

Gene expression signatures associated with immune and virological responses to therapeutic vaccination with Dendritic Cells in HIV-infected individuals

Rodolphe Thiébaut ^1,2,3^ *, Boris Hejblum ^1,2,3^, Hakim Hocini ^3,4,5^, Henri Bonnabau ^1,2,3^, Jason Skinner ^6^, Monica Montes ^6^, Laura Richert ^1,2,3^, Geneviève Chêne ^1,3^, Karolina Palucka ^6^, Jacques Banchereau ^6^, Yves Lévy ^3,4,5^ *

*** Correspondence:** Pr. Rodolphe Thiébaut [rodolphe.thiebaut@u-bordeaux.fr](mailto:rodolphe.thiebaut@u-bordeaux.fr), Pr Yves Levy [Yves.levy@inserm.fr](mailto:Yves.levy@inserm.fr)

# Supplementary table 1. Blood transcription modules (BTM) with a significant change of the abundance over time during the vaccination period.

Description available at:

http://www.interactivefigures.com/meni/btm416_annotation/btmdata/Mx.htm

| Gene Set | Adjusted P value |
| --- | --- |
| blood coagulation (M11.1) | 0.0057800189 |
| cell cycle and transcription (M4.0) | 0.0004940187 |
| enriched in activated dendritic cells/monocytes (M64) | 0.0094216425 |
| enriched in monocytes (II) (M11.0) | 0.0000000000 |
| enriched in monocytes (IV) (M118.0) | 0.0039331490 |
| enriched in neutrophils (I) (M37.1) | 0.0000000000 |
| enriched in neutrophils (II) (M163) | 0.0087753324 |
| enriched in T cells (I) (M7.0) | 0.0030314784 |
| enriched in T cells (II) (M223) | 0.0207772869 |
| formyl peptide receptor mediated neutrophil response (M11.2) | 0.0049225436 |
| immune activation - generic cluster (M37.0) | 0.0000000000 |
| Monocyte surface signature (S4) | 0.0000000000 |
| platelet activation - actin binding (M196) | 0.0094216425 |
| platelet activation (I) (M32.0) | 0.0000000000 |
| platelet activation (II) (M32.1) | 0.0004940187 |
| proteasome (M226) | 0.0062987386 |
| respiratory electron transport chain (mitochondrion) (M238) | 0.0207772869 |
| T cell activation (I) (M7.1) | 0.0207772869 |
| TLR and inflammatory signaling (M16) | 0.0000000000 |
| translation initiation factor 3 complex (M245) | 0.0022230842 |

Not annotated: TBA (M48) p=0.0031493693, TBA (M55) p=0.0059745388, TBA (M153) p=0.0094986325, TBA (M174) p=0.0004940187, TBA (M198) p=0.0165281477, TBA (M211) p=0.0062987386

# Supplementary Table 2. Number of selected genes at each time from the first component of sgsPLS methods (M Sutton, B Liquet, R Thiébaut).

##
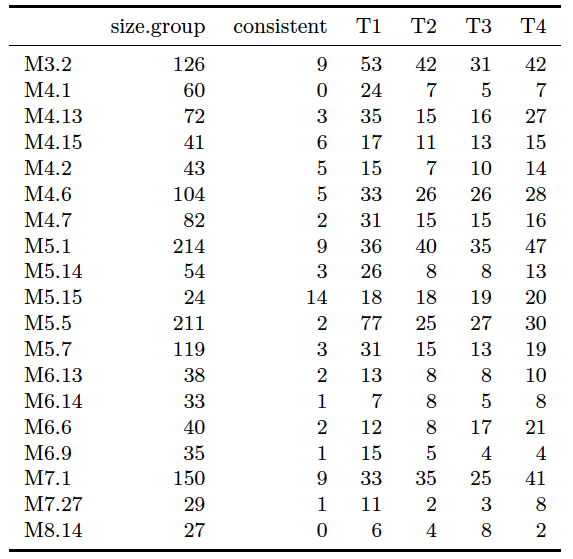


# Supplementary Figure 1. Association between inflammatory gene expression and viral load after antiretroviral interruption. Genes were those selected by sgPLS presented in Figure 5.


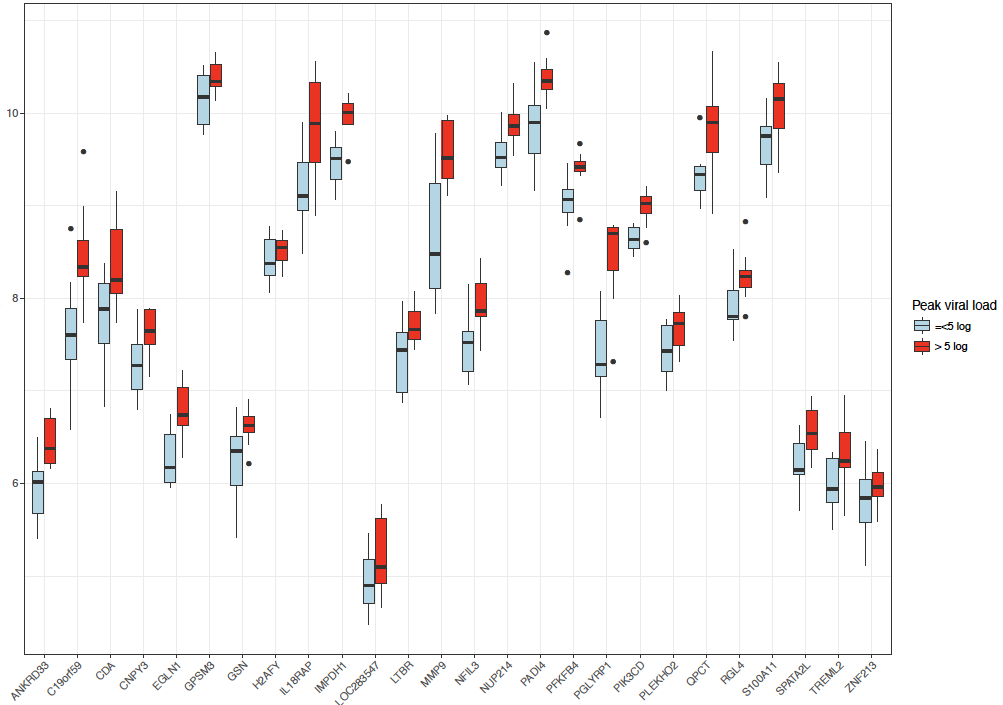

Supplement: Supplementary file 1 [file Data_Sheet_1.docx]
